# Comparison of T_2_*-weighted and QSM contrasts in Parkinson's disease to visualize the STN with MRI

**DOI:** 10.1371/journal.pone.0176130

**Published:** 2017-04-19

**Authors:** Anneke Alkemade, Gilles de Hollander, Max C. Keuken, Andreas Schäfer, Derek V. M. Ott, Johannes Schwarz, David Weise, Sonja A. Kotz, Birte U. Forstmann

**Affiliations:** 1 Amsterdam Brain and Cognition Center, University of Amsterdam, Amsterdam, The Netherlands; 2 Max Planck Institute for Human Cognitive and Brain Sciences, Leipzig, Germany; 3 Epilepsy Center Berlin-Brandenburg, Berlin, Germany; 4 Klinik Haag, Oberbayern/Technische Universität München, München, Germany; 5 Klinik und Poliklinik für Neurologie, Universitätsklinikum Leipzig, Leipzig, Germany; 6 Faculty of Psychology and Neuroscience, Maastricht University, Maastricht, The Netherlands; 7 Netherlands Institute for Neuroscience, an Institute of the Royal Netherlands Academy of Arts and Sciences, Amsterdam, The Netherlands; Henry Ford Health System, UNITED STATES

## Abstract

The subthalamic nucleus (STN) plays a crucial role in the surgical treatment of Parkinson’s disease (PD). Studies investigating optimal protocols for STN visualization using state of the art magnetic resonance imaging (MRI) techniques have shown that susceptibility weighted images, which display the magnetic susceptibility distribution, yield better results than T_1_-weighted, T_2_-weighted, and T_2_*-weighted contrasts. However, these findings are based on young healthy individuals, and require validation in elderly individuals and persons suffering from PD. Using 7T MRI, the present study set out to investigate which MRI contrasts yielded the best results for STN visualization in 12 PD patients and age-matched healthy controls (HC). We found that STNs were more difficult to delineate in PD as reflected by a lower inter-rater agreement when compared to HCs. No STN size differences were observed between the groups. Analyses of quantitative susceptibility mapping (QSM) images showed a higher inter-rater agreement reflected by increased Dice-coefficients. The location of the center of mass of the STN was not affected by contrast. Overall, contrast-to-noise ratios (CNR) were higher in QSM than in T_2_*-weighted images. This can at least partially, explain the higher inter-rater agreement in QSM. The current results indicate that the calculation of QSM contrasts contributes to an improved visualization of the entire STN. We conclude that QSM contrast is the preferred choice for the visualization of the STN in persons with PD as well as in aging HC.

## Introduction

Parkinson’s disease (PD) is characterized by a selective loss of the dopaminergic neurons of the substantia nigra, which causes dysfunctional circuit dynamics within the basal ganglia and the cortex resulting in progressive and severe motor symptoms. STN-substantia nigra projections drive the inhibitory basal ganglia outflow and result in decreased activation of the supplementary motor cortex, which provides a mechanism for bradykinesia developed in PD [1]. Deep brain stimulation (DBS) of the STN provides an effective surgical treatment for PD motor symptoms in advanced stages of the disease, providing instant symptomatic relief [1,2]. However, DBS of the STN is not without risk, and unwanted side-effects have been reported such as apathy, compulsive behavior, hypersexuality, cognitive dysfunction, and clinical depression including suicide, which have been ascribed to the non-motor functions of the STN [2].

Visualization of the STN for DBS is crucial, and STN imaging using Magnetic Resonance Imaging (MRI) is an important part of surgical planning. The STN is small (AP: 5.9mm, ML: 3.7mm, and IS: 5mm as assessed in advanced PD (mean age 54.9 (+/- 8.7) yrs [3]), and the medial border to the SN is particularly difficult to visualize using conventional MRI contrasts. Limitations in STN visualization are reflected in clinical practice. Surgical procedures in many cases complement pre-operative imaging for deep brain surgery with electrophysiological recordings during surgery and the monitoring of immediate clinical effects to ensure correct targeting of the STN. In rare cases electrical stimulators are misplaced and correctional surgery is required to reposition them [4]. It is evident that MRI protocols for optimal visualization of the STN are of crucial importance.

Previous work aimed to optimize STN imaging can be divided according to MRI field strength. Increasing field strengths allow for a more reliable delineation of the STN, and for an improved signal to noise ratio (SNR) proportional to the higher field strength [5]. The clinical potential of ultra-high field strength MRI is recognized within the scientific community [6]. Using ultra-high field fast low angle shot (FLASH) MRI protocols, a number of groups has been successful in visualizing the STN [7–11]. In T_2_ and T2*-weighted images, the STN appears as a hypointense structure [12]. The high iron content of the STN can be exploited to improve STN imaging. Iron causes T_2_* relaxation time shortening and results in a signal reduction in gradient echo magnitude imaging, providing an indirect measure of iron content [12–14]. Iron rich regions show a higher magnetic field perturbation compared to adjacent regions with lower iron content, which can be used to visualize the STN in susceptibility weighted imaging or phase imaging [15–20]. Furthermore, the phase images displaying the magnetic field distribution can be used to calculate Quantitative Susceptibility Maps (QSM). QSM is a novel post-processing technique, which provides a quantitative assessment of the magnetic susceptibility of the tissue under investigation. This is achieved by filtering out the background field contributions, and by resolving the inverse problem arising from field perturbations to magnetic susceptibility [21,22].

It was shown that QSM yield better direct visualizations of the STN than T_2_*-weighted images of phase images [20,23]. The potential merits of the use of QSM in clinical practice are evident, but it is unknown whether the use of QSM contrasts leads to better visualization results compared to T_2_*-weighted images in PD and healthy ageing. We therefore set out to compare QSM and T_2_*-weighted contrasts in 12 persons with PD and a group of 12 age- and gender-matched healthy controls (HC) [10]. Our aim was to determine a superior contrast allowing most reliable segmentation of the STN both in PDs and HCs. For this, manual delineations of the STN were performed by two independent raters, using objective measures including contrast-to-noise ratios (CNR) to determine visibility, and Dice-coefficients to determine inter-rater agreement [24].

## Materials and methods

### Participants

The study was approved by the local ethical committee of the University of Leipzig. Written informed consent was obtained from all participants.

#### Persons with PD

13 persons with a clinical diagnosis of PD according to the British brain bank criteria [25] were recruited from the Department of Neurology at the University of Leipzig. All were right-handed and were diagnosed with mild to moderate disease progression (Hoehn &Yahr: 1–3). Eight of the participants were scanned shortly after their initial clinical diagnosis and were drug naive. Five participants were medicated (Levo-dopa, pramipexol, ropinirol, piripedil, domperidon, ramipril, benserazid, rasigline, omeprazol, amantidine and/or propranolol). One male participant was not included in further analyses because of severe movement artifacts; such artifacts were not observed in any of the other participants. The final analyses include 12 PD participants (mean age = 68 yrs, sd = 9.6 yrs, range = 48–82 yrs, 6 females).

#### Healthy volunteers

12 age- and gender-matched healthy participants, without known history of clinically overt neurological or psychiatric disease, were included as controls. These participants represented a subset of those included in a previous study (mean age = 65yrs, sd = 7.9 yrs, range = 52–77 yrs, 6 females [10]).

### Data acquisition of ultra-high resolution anatomical images

Details on data acquisition have been published elsewhere [10]. Data is available from the Dryad Digital Repository: http://dx.doi.org/10.5061/dryad.t7kp7 and via the NITRC website https://www.nitrc.org/projects/atag_pd/. All participants underwent structural scanning on a 7T Magnetom MRI system (Siemens, Erlangen) using a 24-channel head array Nova coil (NOVA Medical Inc., Wilmington MA). Whole-brain images were acquired with an MP-RAGE sequence [26] (TR = 3000 ms, TE = 2.95 ms, TI = 1100 ms, voxel size = 0.8 mm isotropic, flip angle = 6°, GRAPPA acceleration factor 2) for nine of the age-matched controls and five of the persons with PD; for the remaining age-matched controls and persons with PD, whole-brain images were acquired with an MP2RAGE sequence [27] (HC: TR = 5000 ms, TE = 2.45 ms, TI = 900/2750 ms, voxel size = 0.7 mm^3^, flip angle = 5/3°, GRAPPA acceleration factor 2, PD: TR = 5000 ms, TI1 = 900 ms, T_2_ = 2700 ms, TE = 2.45 ms, voxel size = 0.7mm isotropic, flip angle = 5/3°, GRAPPA acceleration factor 2).

**Fig 1:**
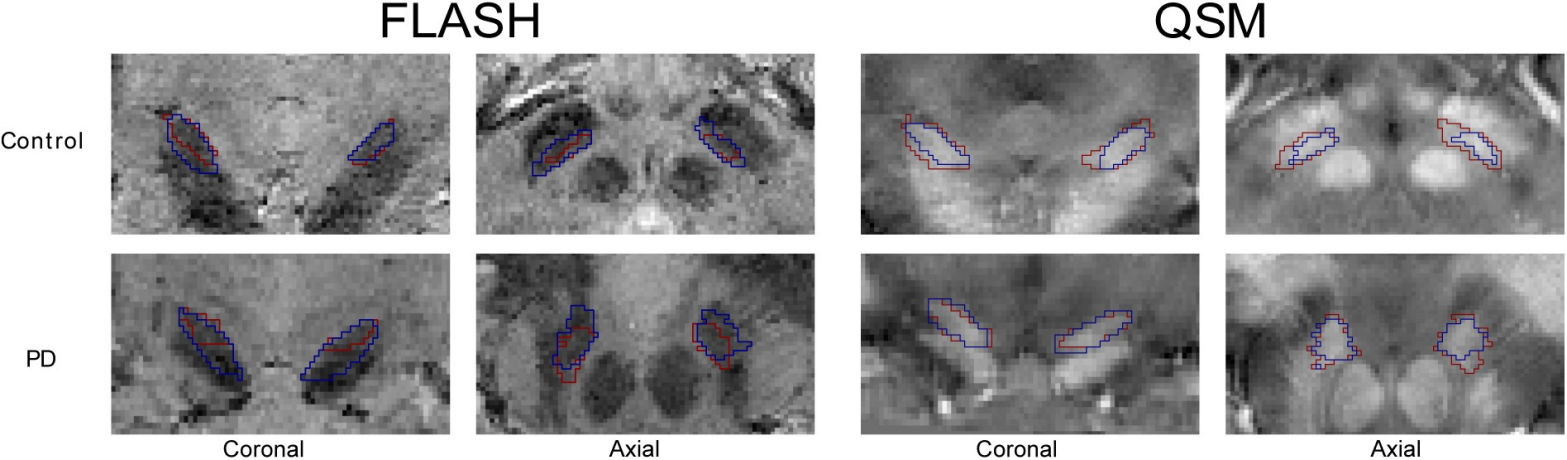
STN outlines. Representative views of the STN (coronal and axial views) of the T_2_*-weighted and QSM contrasts. Note the overlapping outlines of the STN masks made by two different raters in red and blue.

Further, a multi-echo spoiled 3 dimensional (3D) gradient echo (FLASH) sequence [28] (HC: TR = 43 ms, TE1 = 11.22 ms, TE2 = 21.41 ms, TE3 = 31.59 ms, flip angle = 13°, voxel size = 0.5x0.5x 0.6 mm, 56 coronal slices; PD: TR = 40 ms, TE1 = 9.76 ms, TE2 = 19.19 ms, TE3 = 28.62 ms; flip angle = 12°; voxel size = 0.6x0.6x0.8 mm, 88 coronal slices) was acquired. Total acquisition time was approximately 60 min broken down into: FLASH 14:20min (HC), or 16:40 min PD, MPRAGE 6:44 min, MP2RAGE 10:57 min. oil combination of the phase images of the FLASH data were done automatically using the scanner vendor software (version VB17) and can result in some minor phase singularities. The masking of the data was done by creating a binary mask using brain extraction protocol (BET). The masked phase data, which show the field perturbations of a magnetic susceptibility distribution, were unwrapped using a best-path 3D unwrapping algorithm [29]. Subsequently, the unwrapped phase data were converted in units of ppm, the minor phase singularities, and background phase contributions were reduced using the sophisticated harmonic artifact reduction for phase data (SHARP) algorithm [30]. To calculate the magnetic susceptibility distribution from filtered phase data, the Superfast Dipole Inversion (SDI) approach was used. The convolution kernel threshold for the SDI was [delta] = 2/3 and is based on previous work [31]. See Fig 1 for a representative T_2_* and QSM image.

We would like to point out that UHF MRI can potentially show larger geometric distortions as compared to lower field MRI [6]. Previous work by Cho et al. [32] has demonstrated that on 7T MRI, using a bandwidth larger than 40 kHz combined second-order shimming results in negligible geometric distortions of “less than a submillimeter”. We apply a bandwidth of 44.8kHz, and therefore geometric distortions are kept to a minimum. Additionally, based on the work of Dammann et al. [33] distortions in the center of the volume are minimal [34].

### Manual segmentation of the STN

Manual segmentation was performed in individual space using either the FLASH or QSM volumes using the approach described in [10,35]. The opacity of the individual FLASH magnitude images corresponding to the three echoes was reduced such that each contributed 33% of the pixel intensity. Contrast settings were adapted to suit the individual computer monitors used by the raters. To compare the location of the STN, the conjunction masks were registered to standard 0.5mm MNI space. The average of the three TE FLASH volumes were registered linearly to the MP(2)RAGE volumes with FLIRT using 6DoF, mutual information, and trilinear interpolation as implemented in FSL 5.0.9 [36]. The linear whole brain MP(2)RAGE registration to MNI space was done using FLIRT using 12DoF, correlation information, and trilinear interpolation. All volumes were skull stripped prior registration using a brain extraction protocol (BET) [37].

All registration steps were individually checked by visual inspection for gross misalignments. To complete the registration process, the FLASH and QSM STN masks were transferred to standard space using the resulting transformation matrices and nearest neighbor interpolation.

### Calculations

#### STN volume

STN size was calculated in individual space as the conjunct volume of the masks of both raters as described previously [10]. Only voxels that were included in the STN by both individual raters were included in the conjunct mask.

#### Dice coefficient

Inter-rater reliability was calculated using a Dice-coefficient [24]. Dice-coefficients were compared between MRI contrasts as well as between PD and HC participants.

The Dice coefficient was calculated as follows:

$$D=\frac{2\times |m_{1}\cap m_{2}|}{\left|m_{1}|+|m_{2}\right|}$$

Where |*m*_*i*_| is the size of mask *i* and |*m*_1_ ∩ *m*_2_| is the size of the conjunct mask of mask 1 and 2. A conjunct mask of a set of masks M only includes voxels included in the STN by both raters.

#### Center of mass

For each individual mask, the center of mass was calculated in individual space. Within participant and modality, a mean center of mass was calculated over the two raters, as well as the mean distance between the mean center of mass and the centers of mass of the individual rater masks. This was a measure of inter-rater agreement on the position of the STN. Additionally, the center of mass was calculated for each conjunction mask in standard MNI space to test whether there was a difference in STN location in PD compared to HC participants. As there were no a-priori hypotheses on hemisphere the absolute values for x-coordinates for the left hemisphere were used allowing comparison between the x-coordinates between the left and right hemispheres.

#### Contrast to noise ratio (CNR)

The CNR was calculated in individual space to measure the contrast values at the outer borders of the mask of the STN to the surrounding structures for both T2* and QSM conjunct masks. A higher CNR value would contribute to a more precise delineation of the STN. The CNR was calculated as follows:

$$CNR=\frac{S_{I}-S_{O}}{\sigma_{0}}$$

S_I_ represents the signal in the STN, as calculated by the mean value of all the voxels in the conjunct mask (voxels that were scored inside the STN by both raters). S_O_ is the signal outside the STN, calculated as the mean value of all voxels that directly border the outside of the STN disjunct mask (all voxels scored inside the STN a single rater). *σ*_0_ is the standard deviation of the set of QSM intensities in these voxels. This approach was adopted to ensure that outside voxels were not part of the separate masks of the individual raters STN.

#### QSM values

QSM values were calculated for each conjunct mask in individual space. All values were normalized by subtracting the QSM values obtained from the control region, i.e., the average values from the cerebrospinal fluid (CSF) obtained from the left and right lateral ventricle, and which appeared homogenous based on visual inspection, from the conjunct STN masks. The normalization of the QSM values was necessary due the use of high pass filtering and the use of a single echo to calculate the QSM values [19,31]. Mean QSM values in the STN were calculated for individual conjunct masks. Values were not compared across groups in view of slight differences in MR parameters.

#### Statistical analyses

An IPython Notebook environment with R for statistical inferences was used for statistical analyses [38,39]. In view of lateralization reports in PD as well as STN function [40–42], differences in STN size between hemispheres were tested for. Paired t-tests were used to test for differences in STN size between the left and right hemisphere in the PD participants.

The dependent variables STN volume, Dice coefficient, difference in center-of-mass between raters, and CNR were tested using a two-way ANOVA (group and MR contrast). For the creation of confidence intervals we used the classical bootstrap procedure, using random sampling with replacement, as implemented in the Seaborn statistical plotting library [43,44].

## Results

### STN volumes

Paired t-tests showed that there was no difference in STN size between the left and right hemisphere in the PD participants (t(11) = -1.97, p = 0.07 for QSM; t(11) = 0.45, p = 0.65 for T2*). There was also no difference in the controls (t(11) = 2.03, p = 0.07 for QSM; t(11) = 1.58, p = 0.14 for T2*-weighted). As no differences across hemispheres were observed, the data from both hemispheres were collapsed using the averaged values for subsequent analyses. Statistical analyses did not reveal any differences in STN volume between the PD and the control group nor an interaction between groups and contrast. There was a significant difference in STN volume between QSM maps and T_2_*-weighted (two-way ANOVA, F(1, 44) = 6.08, p = 0.018 for QSM vs. T_2_*-weighted, F(1, 44) = 1.72, p = 0.20 for PD vs. matched controls, and F(1, 44) = 0.23, p = 0.63 for the interaction modality*group; see Table 1).

**Table 1:** Main results for T_2_*-weighted and QSM contrasts.

		T_2_*	QSM
STN Volume (mm^3^)	PD (n = 12)	57.4 (14.3)	76.8 (21.6)
	Controls (n = 12)	69.2 (25.9)	82.34 (27.6)
Dice coefficient	PD	0.76 (0.10)	0.87 (0.04)
	Controls	0.84 (0.03)	0.88 (0.03)
Distance between Centers-of-mass of the two raters (mm)	PD	1.45 (0.64)	0.68 (0.42)
	Controls	1.01 (0.38)	0.57 (0.26)
Contrast-to-noise ratio (CNR)	PD	0.48 (0.13)	0.59 (0.14)
	Controls	0.50 (0.06)	0.59 (0.11)
Mean normalized QSM value	PD		0.0256 (0.0379)
	Controls		0.0711 (0.0401)

Data are presented as mean (std). STN = subthalamic nucleus, QSM = Quantitative Susceptibility Mapping

### Dice coefficients

**Fig 2:**
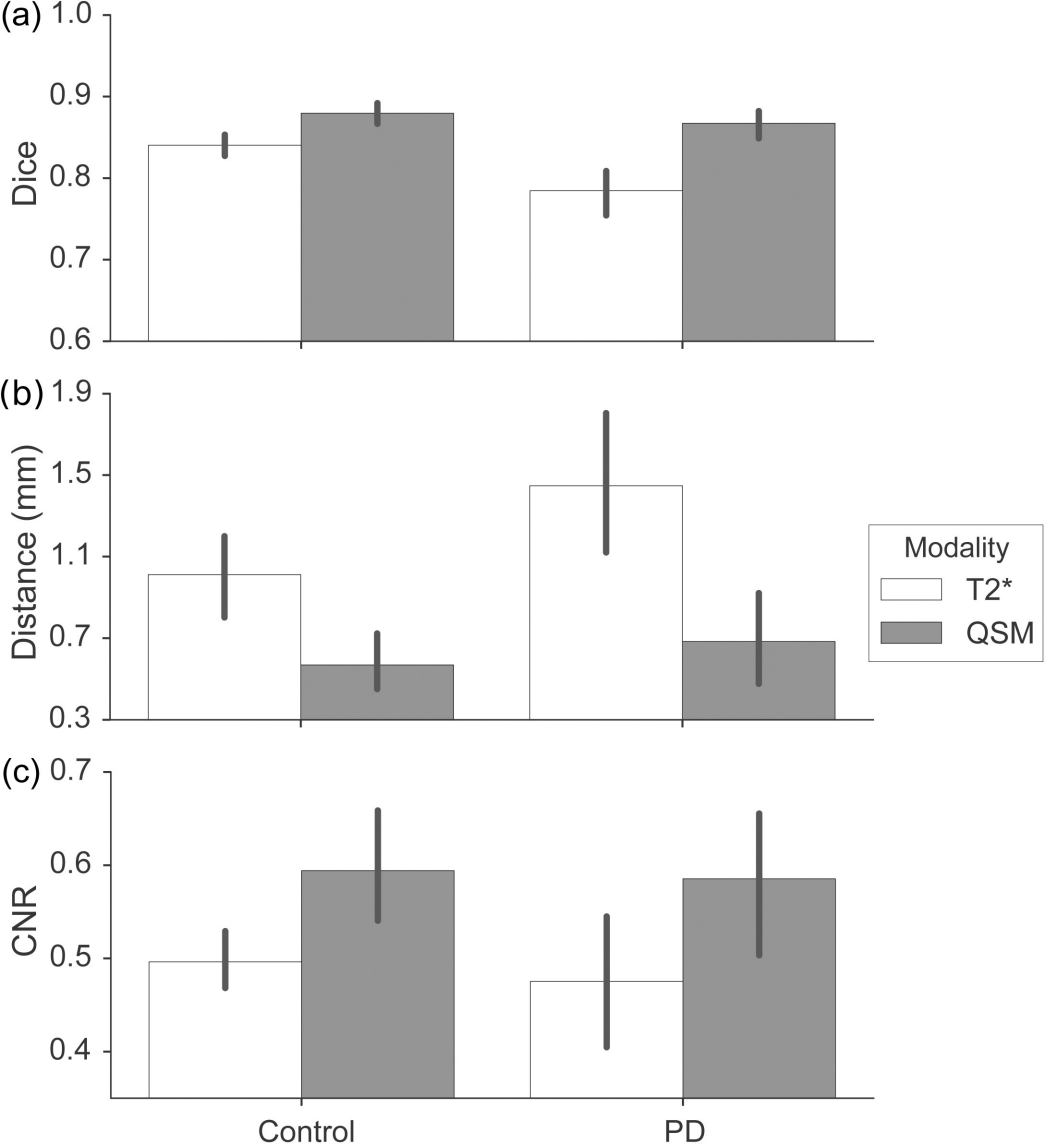
Quantitative results. A) Mean dice-coefficients. PD participants are compared to healthy controls. Error bars indicate 95% bootstrapped confidence intervals. Note the significant increase in Dice-coefficient in QSM contrasts. B) Mean distances between the center of mass. Error bars indicate 95% bootstrapped confidence interval. Note the smaller distances in the QSM corresponding to higher agreement between raters. C) Average contrast-to-noise ratios (CNRs). Error bars indicate 95% bootstrapped confidence interval. Note that higher CNR-values in QSM contrasts reflect improved visibility.

Dice coefficients for all STNs (left and right, PD, and controls) in both contrasts were calculated. Comparison of Dice-coefficients between QSM and T_2_*-weighted contrasts revealed that inter-rater agreement was significantly higher in the QSM contrast as compared to the T_2_*-weighted contrast (F(1,44) = 19.67, p = 0.0001). Additionally, Dice-coefficients were significantly lower in persons with PD as compared to HC (F(1,44) = 19.67, p = 0.0078). Furthermore, there was a significant interaction between the used contrast and group (F(1,44) = 4.10, p = 0.049) indicating a larger increase in the Dice coefficient for QSM vs. T_2_*-weighted in PD as compared to HC (effect size of .066, see Table 1, Figs 1 and 2A).

### Center of mass

All individual values for center-of-mass were calculated and, in addition, the distance to the center-of-mass was calculated for each mask in both individual raters. The agreement between raters was measured by the mean distance between their masks. Agreement was higher in individually segmented QSM masks as compared to T_2_*-weighted masks, based on the FLASH images as reflected by a smaller distance (F(1, 44) = 21.92, p = 0.00003). The distance was also significantly smaller in HC as compared to the PD participants (F(1,44) = 4.56, p = 0.038). No significant interaction was observed (F(1,44) = 1.55, p = 0.22; see also Table 1, Fig 2B). The difference between QSM and T_2_*-weighted could not be ascribed to a shift into a single specific direction.

There was no significant difference in the center-of-mass in standard space between the two groups in all three spatial directions for the T_2_*-weighted masks (x-axis: t(45.23) = -1.06, p = 0.89; y-axis: t(45.93) = 1.06, p = 0.89; z-axis: t(37.31) = -2.32, p = 0.08; p-values are Bonferroni corrected). The QSM masks of the PD participants were however on average located 0.96mm more medial and 1.73mm more inferior in standard MNI space (x-axis: t(45.44) = -2.51, p = 0.047; y-axis: t(43.72) = 1.38, p = 0.53; z-axis: t(41.01) = -2.86, p = 0.02; p-values are Bonferroni corrected).

**Fig 3:**
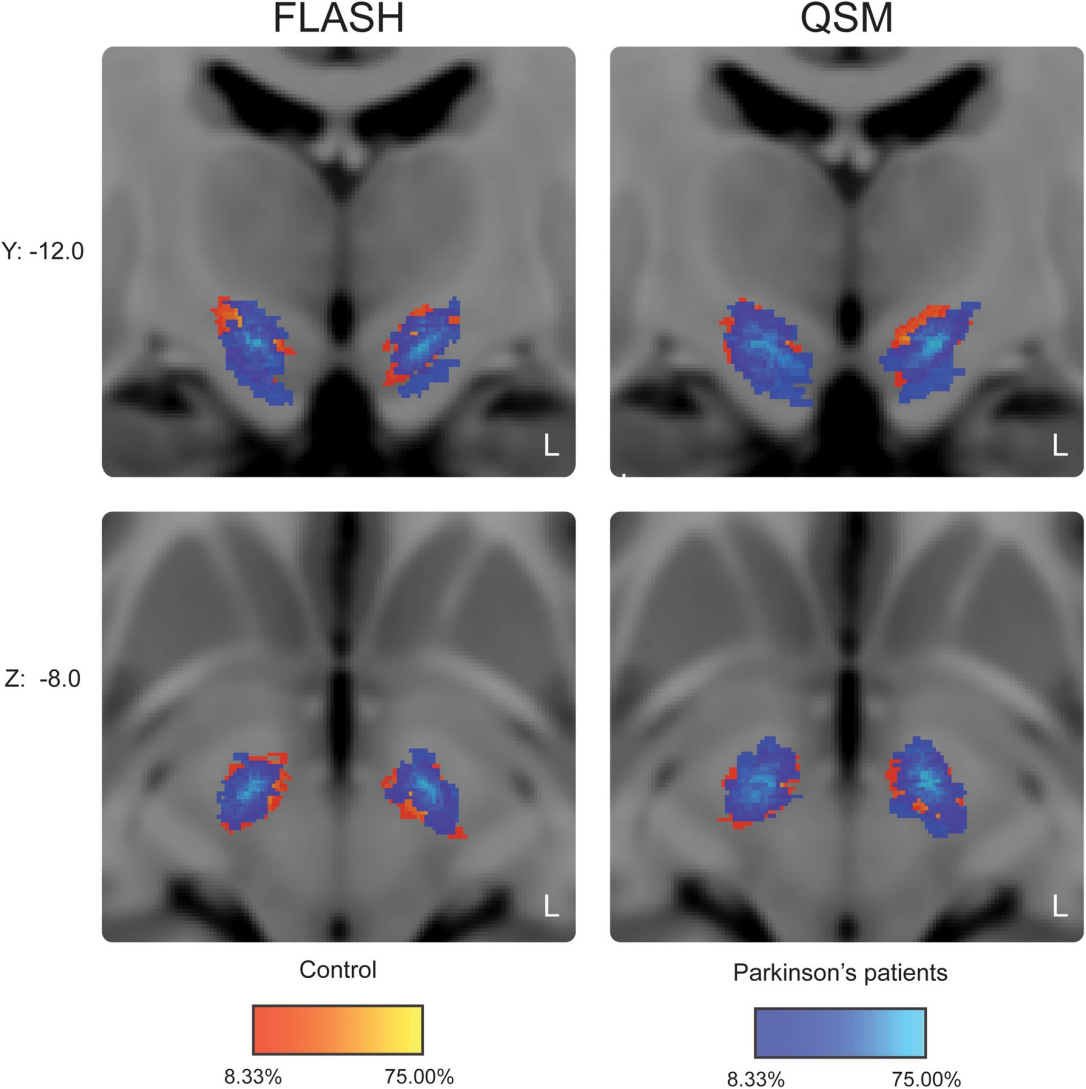
Probabilistic STN atlas in standard MNI space. The probability maps for the controls are in red-yellow. Superimposed in blue are the probability maps for Parkinson’s disease patients. Color intensity reflects the percentage overlap between individuals.

The average percentage overlap in the location of the STN was 52% in PD and 71% in HC participants. See Fig 3 for the overlap between groups in standard MNI space.

### Contrast-to-noise ratio (CNR)

The CNR was calculated to provide an objective measure for the difference in the visibility of STN borders in QSM and T_2_*-weighted contrasts. For the T_2_*-weighted contrast, contrast-to-noise was largely comparable across the three echo times: in the HC group, CNR was 0.42 (std. 0.09) for the first echo time, 0.52 (std. 0.07) for the second echo time, and 0.56 (std. 0.07) for the third echo time. In the PD group, CNR was 0.41 (std. 0.19) for the first echo time, 0.50 (std. 0.17) for the second echo time, and 0.53 (std. 0.14) for the third echo time. Because the STN masks were drawn on an average of all three echo times, the three corresponding CNR values were also averaged before comparison with the CNR of the QSM images. CNR was higher in QSM as compared to the T_2_*-weighted images (F(1,44) = 10.32, p = 0.002). There was no main effect of group (F(1, 44) = 0.21, p = 0.65), and no interaction between MR contrast and patient group (F(1, 44) = 0.04, p = 0.85; see also Table 1 and Fig 2C).

## Discussion

Today DBS is an established safe and effective treatment for a variety of movement disorders [4]. Electrode placement for DBS remains challenging and requires a high degree of precision. In preparation for the surgery, the target is defined using MR images, and during surgery using microrecordings and macrostimulation [45]. Neuroimaging is therefore crucial for successful DBS surgery, and surgical outcomes are likely to benefit from further optimalization of visualization techniques of the STN.

T_2_*-weighted contrasts are commonly used for the visualization of deep brain structures. This study was designed to compare a T_2_*-weighted contrast to the relatively new QSM contrast. The precision with which the STN can be delineated was determined. Comparison of T_2_*-weighted and QSM showed that QSM contrasts provided a higher level of precision: Dice coefficients increased on average from 0.76 (T_2_*-weighted) to 0.87 (QSM) in the PD group, and from 0.84 to 0.88 in the sex and age-matched controls. The effect was observed in both groups despite minor differences in the 3D FLASH sequences. We chose a previously published method to calculate QSM values. In view of the ongoing developments in the field, it is likely that the image quality can be improved even further in the future. These findings are in line with previous qualitative assessments using T_2_*-weighted angiography [46]. Although we did not investigate other contrasts across our current studies, it is possible that other contrasts such as quantitative T_2_*-/R_2_*-maps may also provide a higher level of precision than raw T_2_*-weighted magnitude images, although, at 3T, they do not allow visualization of the SN-STN border [47]. For this reason we did not perform comparisons of quantitative MR parameters between groups.

Our results show an average difference in the location of center-of-mass between two raters of 1.44 mm in PD participants for the T_2_*-weighted contrast, whereas this deviation was less than half (0.68 mm) in the QSM contrast. The differences observed in the control group were comparable, despite differences in the imaging parameters used between the groups. Additionally, the conjunct masks were smaller in T_2_*-weighted as compared to QSM maps. The observed smaller volume of the T_2_*-weighted masks was related to the lower inter-rater reliability. In view of the small size of the STN, the magnitude of these deviations is important. The precision gained by the analyses of QSM images was present in all directions and could therefore not be exclusively ascribed to the STN border with the SN, a border that is challenging to visualize. The results may, in part, be explained by improved CNRs. Our findings indicate that QSM provides an improved contrast of the STN as compared to T_2_*-weighted images. The current findings have a number of important implications. For clinical practice it is crucial that the surgical target for deep brain stimulation can be accurately localized in individual space.

We would like to point out that the scanning protocol as described in the present study was designed for research and not clinical purposes. The total acquisition time of approximately 1h as described in the present study would therefore not be suitable in a clinical setting. We would like to note though that in addition to the FLASH (~14 min), other sequences were also acquired. Acquisition of a FLASH sequence suitable for QSM calculations within a clinical setting is therefore feasible. A number of neurosurgical teams report specific targeting of the dorsolateral part of the STN [48,49]. Differences in STN volume based on the MR contrast could directly affect the target location in the dorsolateral STN and thereby surgical planning.

Our probabilistic maps provide a first indication of the location of the STN in MNI space. Limited overlap within both groups (maximally 58% of the STNs of the patients were represented in individual voxels) indicated substantial variation in STN location, which could be partly attributed to age-effects [50]. The QSM contrast provides improved image quality for the visualization of the PD STN, which may be relevant for surgical procedures. In basic research, more precise delineations of the STN using QSM are important to study inter-individual differences in both function and structure.
